# Enhancing Resourcefulness to Improve Outcomes in Family Caregivers and Persons with Alzheimer's Disease: A Pilot Randomized Trial

**DOI:** 10.1155/2014/323478

**Published:** 2014-09-29

**Authors:** Elizabeth W. Gonzalez, Marcia Polansky, Carol F. Lippa, Laura N. Gitlin, Jaclene A. Zauszniewski

**Affiliations:** ^1^Doctoral Nursing Program, Division of Graduate Nursing, College of Nursing and Health Professions, Drexel University, 245 N. 15th Street, MS 1030, Philadelphia, PA 19102-1192, USA; ^2^Department of Epidemiology and Biostatistics, Drexel University School of Public Health, Philadelphia, PA 19104, USA; ^3^Memory Disorder Clinic, Department of Neurology, Drexel University College of Medicine, Philadelphia, PA 19102-1192, USA; ^4^Center for Innovative Care in Aging, Department of Community-Public Health, Johns Hopkins School of Nursing, Baltimore, MD 21205, USA; ^5^Frances Payne Bolton School of Nursing, Case Western Reserve University, Cleveland, OH 44106-7123, USA

## Abstract

This pilot randomized trial tested an intervention aimed at enhancing resourcefulness in family caregivers of persons with dementia, postulating that caregivers' emotional outcomes (anxiety and depression) and role outcomes (reward, strain, mutuality, and preparedness) would be improved, and problem behaviors in the care recipients (persons with dementia) would be reduced as a result of the intervention. Subjects were stratified by race (white or African American) and by baseline resourcefulness (high or low). Family caregivers were randomly assigned to an intervention group in which subjects attended six resourcefulness training sessions, meeting for 2 hours weekly over 6 weeks, or to a control group that received no treatment. Small to medium effects were shown for the intervention program on resourcefulness, anxiety, and preparedness of the caregivers and on frequency of behavior problems in the care recipients. Caregivers in the intervention group reported significantly more resourcefulness skills, with a medium effect at week 6 and a small effect 12 weeks later, compared with the control group. Persons with dementia had fewer behavior problems in the intervention group compared with control, although the difference was not significant. Caregivers' anxiety was reduced in the intervention group at 12 weeks.

## 1. Introduction

In today's healthcare system, as the environment for care shifts to maintaining older adults in the community, more families are assuming responsibility for the care of elderly family members at home. The importance of families as a healthcare resource is predicted to significantly increase during the next century.

Families often take on the responsibilities of caring for sick or disabled elderly family members at home with minimal or no preparation or knowledge about the person's health problems. Caring for a person with Alzheimer's disease (AD) is particularly demanding as the needs for care escalate as the disease progresses [1]. Family caregivers may also experience stress because their family roles are changing; they face the prospect of losing a family member [2]; and they may feel unprepared to meet caregiving demands [3]. Studies have indicated that family caregivers of persons with dementia are at risk for depression, poor health, diminished quality of life, and increased mortality [4–6]. Family caregivers often feel powerless about their ability to influence their family member's recovery because AD is debilitating and fatal. Despite these potential adverse effects, some family caregivers view certain aspects of caregiving in a positive light and handle the stresses associated with caring for a family member with dementia with little difficulty [7–10]. Some families report that being involved in the care of a person with dementia results in an increased sense of purpose [11].

Researchers have shown that family caregivers of persons with dementia have multiple needs, which include but are not limited to acquiring information about the health issues of the person with dementia; developing problem-solving skills so that they may help to achieve positive outcomes for the care recipient as the disease progresses [12]; learning strategies to prevent and manage behavioral symptoms; learning to communicate effectively with the person with dementia and others; and planning for the future [13].

Interventions to alleviate distress among family caregivers of persons with dementia include three overarching, empirically based treatments: (1) psychoeducational skill building that focuses on increasing caregiver knowledge of a specific disorder and/or teaching the caregiver specific skills; (2) specific forms of individual or group therapy or counseling (e.g., cognitive-behavioral or psychodynamic); and (3) two or more conceptually different approaches integrated in a single intervention package, such as combining skill training with a support group or with family counseling.

Buckwalter et al. [14] implemented a one-to-one home health nurse-delivered instructional program teaching behavior management to family caregivers in the home setting using the “progressively lowered stress threshold” theoretical framework. They demonstrated that the program reduced caregiver burden and depression. Gitlin and associates [15] developed an occupational therapy-based skill-building program for home caregivers that demonstrated sustained positive effects on caregiver skills and affect. Ostwald and associates [16] have described a group psychoeducational program that resulted in reduced depression and burden among caregivers. Hepburn et al. have reported on a psychoeducational program on decision-making training that showed reduced caregiver distress [17]. They also reported on the Savvy Caregiver Program, a psychoeducational program delivered in different sites in the United States, which increased caregiver competence and mastery and reduced distress [18].

Interventions that combine education and an individualized counseling program have shown success in delaying institutionalization of persons with AD [19]. A national multisite program (Resources for Enhancing Alzheimer's Caregiver Health II) examined the effects of a multicomponent intervention using skills training, education, and telephone support, in a study including 600 black, Hispanic, and white/non-Hispanic white participants. This study showed significant benefit to most caregivers, but not all caregivers achieved the same results. Specifically, nonspouse black/African American caregivers did not benefit as much as other groups [20]. This finding indicates that additional study is needed to determine who benefits most from which type of intervention. Most earlier intervention studies in caregiving have focused on stress and caregiver deficits.

This study was designed to address the gap in the current state of intervention research with family caregivers of persons with dementia. Specifically, the present study targets caregivers' resourcefulness, a protective factor that may buffer the effects of chronic stress on caregivers' health. Rosenbaum [21] defined learned resourcefulness as a repertoire of coping strategies to control and manage internal cognitive and emotional responses to perceived stressors. Resourcefulness is linked to self-control skills, which are related to cognitive-behavioral strategies designed to manage stressful circumstances. Techniques include cognitive reframing, problem solving, and the regulation of emotions and cognitions. Interventions to enhance resourcefulness in family caregivers have multiple components involving psychoeducational skill building to increase knowledge about AD; teaching specific skills such as problem solving; teaching skills to manage stress and deal with the patient's behavioral problems; and group support. The development of the intervention reported on here was guided by three theoretical approaches: Rosenbaum's conceptual model of learned resourcefulness, Johnson's self-regulation theory, and Burr's interactional role theory.

Self-regulation theory postulates that an individual's cognitive schema of an impending stressful event is instrumental to processing information as the event unfolds and to guiding responses (emotional outcomes) and behaviors (functional outcomes) during the event [22]. The cognitive schema is defined as an image or picture in the mind of an event or situation that includes knowledge about what is happening in the event, as well as plans on how to deal with the event in order to achieve the desired outcomes. Providing information about dementia will facilitate the caregiver's forming a clear, unambiguous schema about what to expect as the disease advances in the person with dementia.

Anticipatory preparation for new roles is a key concept in Burr and colleagues' [23] interactional role theory related to family processes. The theory provides insight about how to assist family caregivers with effective role implementation. Roles are defined as goal-oriented patterns of behavior [24], and interaction between role partners is emphasized [25]. Archbold et al. [26] applied Burr's work to family caregiving for elders in the home setting and developed a nursing intervention program (PREP, for “preparedness, enrichment, and predictability”) that uses a mutual agreement method. In this program, caregivers sign a contract with the nurse to work on two problem areas of greatest concern, caused by the patient's condition. Then specific information for each problem was provided and list of suggested care activities that caregivers could select based on their preference [27]. Building on the work of Archbold and associates, a mutual agreement method was developed based on role theory's assumptions that how well a caregiver performs in a role is associated with clarity of role expectation. An easier role adaptation is associated with well-defined role tasks.

In summary, based on self-regulation theory, we postulated that providing educational information about AD to family caregivers would facilitate the formation of a clear cognitive schema that would strengthen caregivers' understanding about disease-associated changes as well as their ability to interpret these changes and adapt to them. Subsequently, understanding disease-related changes, cognitively reframing issues, and learning problem-solving skills might help caregivers moderate their responses, leading to less frustration, anxiety, or depression. We also postulated that as a result of improved emotional outcomes and strengthened role outcomes for caregivers, outcomes for the person with dementia, such as frequency of behavior problems, would be reduced.

The purpose of this study was to evaluate the effects of a multicomponent intervention that included teaching resourcefulness and providing group support on outcomes of both persons with AD and their family caregivers. We examined the following research questions: what are the effects of the intervention program on family caregivers' emotional outcomes (anxiety, depression) immediately (6 weeks) after the intervention and 12 weeks after intervention? What are the effects on caregivers' role outcomes (reward, strain, mutuality, and preparedness) immediately (6 weeks) after the intervention and 12 weeks after the intervention? Finally, what are the effects on frequency of behavior problems in the care recipient, immediately (6 weeks) after the intervention and 12 weeks after the intervention?

## 2. Method 

We conducted a randomized clinical trial using the stratification variables of race (white versus African American) and resourcefulness at baseline (high or low). Stratification by race was based on previous study [28] that showed African American caregivers are more resourceful than white caregivers. Family caregivers were randomly assigned to the intervention or control group. Outcomes were measured at baseline, 6 weeks (intervention endpoint), and 12 weeks after the intervention.

### 2.1. Sample

The stratified random sample included 102 family caregivers (50 experimental and 52 control subjects) who lived with and provided care to a person with AD. To be included, women family caregivers had to be living with a person diagnosed with AD and providing at least 8 hours of care for the person weekly; had to be able to read, understand, and speak English; and had to be cognitively intact, as determined by a score of at least 7 of 10 on the Short Portable Mental Status Questionnaire [29]. Potential participants with family members diagnosed with probable AD were referred to the study by a neurologist research team member (CFL) and called the principal investigator (EWG) if they were interested in participating. A neurologist (CFL) who isa coinvestigator verified that patients had a probable diagnosis of AD using the NINCDS/ADRD criteria for probable AD [30]. Patients were excluded if they were bed bound or had Parkinson's disease, multi-infarct dementia as a primary diagnosis, schizophrenia, or bipolar disorder.

### 2.2. Procedures

Following approval for protection of human subjects and informed consent procedures, all participants signed a consent form. Data were collected during individual, face-to-face, structured interviews conducted by trained data collectors in a private setting at a mutually agreed upon time. During the initial interview, information on demographic characteristics (age, gender of patient, and race) and type of health conditions was obtained for both caregivers and care recipients, and the caregivers completed structured measures of the study variables. Baseline data on level of and race (African Americans; whites) were used to randomly assign caregivers to the control (standard care) or the intervention group. Beginning within 2 weeks of the initial interview, subjects met in groups of five or seven for 2 hours weekly, over the course of 6 weeks. Within a week after the intervention concluded, participants were interviewed a second time, and a third interview took place 12 weeks after the intervention concluded. The study examined the effects of enhancing resourcefulness skills on family caregivers' emotional outcomes (decreased anxiety, depression), role outcomes (increased preparedness, reward, mutuality; decreased role strain), and care recipient outcomes (decreased frequency of behavior problems).

### 2.3. Measures

Demographic information including age, gender, ethnicity, type of relationship (spouse versus nonspouse), education, employment status, marital status, annual household income, and health conditions for family caregivers and care recipients, and clinical variables were obtained. The instruments used to measure the study variables have been supported as valid and reliable in prior work. Primary outcome measures include caregiver anxiety, depression, and resourcefulness. Secondary outcome measures include caregiver preparedness, role reward, caregiver strain, quality of caregiver-care recipient relationship (mutuality), and frequency in occurrence of behavior problems.

#### 2.3.1. Primary Outcomes

The State-Trait Anxiety Inventory, A-State (STAI) [31], a 20-item tool, was used to measure family caregivers' current anxiety level. For this sample, internal consistency reliability for the scale was 0.93.

Depression was measured using the Center for Epidemiologic Studies Depression Scale (CESD) [32], a 20-item self-report scale that represents a symptom cluster consisting of negative affect, positive affect, interpersonal problems, and somatic activity. Caregivers respond to 20 statements (based on feelings during the past week) using the response categories 0 (rarely or none), 1 (some or a little), 2 (occasionally or moderate), and 3 (most or all). Total scores range from 0 to 60 with higher scores indicating more depressive symptoms. The CES-D scale has been widely used with older adults, demonstrating good internal consistency with Cronbach's alphas in the 0.86 to 0.92 range in caregiving samples [33]. For this sample, internal consistency reliability for the scale was 0.91.

Resourcefulness was measured using the Self-Control Scale (SCS) [21]. The SCS consists of 36 Likert-type items. Participants indicate the degree to which each item describes their behavior on a 6-point scale, ranging from 3 (very much like me) to −3 (not at all like me). Scores may range from −180 to +180; higher composite scores, after reverse scoring of 11 items negatively phrased, indicate greater resourcefulness. Internal consistency estimates ranging from 0.75 to 0.85 have been reported in studies with older adults [29, 34, 35]. For this sample, internal consistency reliability for the scale was 0.83.

#### 2.3.2. Secondary Outcomes (Family Caregivers' Role Outcomes)

Preparedness, defined as a caregiver's perceived readiness to provide care, was measured using the Preparedness for Caregiving Scale (Stewart & Archbold, 1994, Family Caregiving Inventory, unpublished). The family caregiver rates each of the nine items on a 5-point scale that ranges from 0 (not at all prepared) to 4 (very well prepared). The summed scored has a possible range from 0 to 36, with higher scores indicating higher preparedness levels. An example of an item on the Preparedness for Caregiving Scale is “How well prepared do you think you are to take care of your family member's emotional needs?” Internal consistency reliability for the scale was 0.84 in this sample.

Family caregivers' role reward was measured using the Family Role Reward Scale (FRRS) (Stewart & Archbold, 1994). Family caregivers rate each of the items on a 5-point scale that ranges from 0 (not at all) to 4 (a great deal). An example of an item on the FRRS is “Does caring for your family member allow you to preserve his/her integrity?” Cronbach's alpha was 0.93 in this study.

Family caregiver strain was measured using the Caregiver Role Strain global strain subscale (CRS) (Stewart & Archbold, 1994). Caregivers' role strain refers to difficulty experienced in fulfilling the caregiving role. Family caregivers rate each item on a 5-point scale that ranges from 0 (not at all) to 4 (a great deal). An example of an item on the CRS is “How often would you say that taking care of your family member is very difficult?” Cronbach's alpha was 0.86 in this study.

The quality of the relationship between the caregiver and the care recipient was measured using the Mutuality Scale (MS) (Stewart & Archbold, 1994). Family caregivers rate each of the 14 items on a 5-point scale that ranges from 0 (not at all) to 4 (a great deal). Scores on the summed scale range from 0 to 60. An example of an item on the MS is “To what extent do the two of you laugh together?” Cronbach's alpha was 0.92 in this sample.

#### 2.3.3. Secondary Outcome: Patient Outcomes

Behavior problems were measured using the 24-item Revised Memory and Behavior Problem Checklist (RMBPC) [36]. This instrument measures the frequency of the care recipient's problem behaviors in the following domains: disruptive behavior (wandering, aggression), memory-related behavior (repeating questions and stories), and depression. Higher scores indicate more frequent occurrence of behavior. Cronbach's alpha was 0.84 in this sample.

### 2.4. Intervention Group

Family caregivers participated in six group sessions of resourcefulness training, in groups of five to seven caregivers who met for 2 hours weekly. A modified version of the resourcefulness training developed by Zauszniewski (personal communication) and the problem-solving training developed by Kurylo and associates [37] were used. The training was a short-term, structured, time-limited intervention that taught and reinforced the cognitive-behavioral skills constituting problem identification, coping strategies, problem solving, priority setting, and decision making. The training includes six modules using FOCUS as an acronym for ease in recalling the components (focus, optimism, creativity, understanding, and solution). FOCUS is implemented in the following manner. The first component involves description of a problem identified in a card-sorting procedure based on the Revised Memory Problem Checklist. The caregiver is asked to rank-order concerns or problem from the most important to the least important. Family caregivers are asked to select two problem areas to focus on in providing care based on their own preferences and on the abilities of the person with dementia.

Caregivers are guided in “finding the fact” (“F” in FOCUS) about the identified problem. The caregiver is then assisted in articulating a specific attainable goal for the most important problem. The second component is to assist the caregiver in developing a sense of “optimism” (“O” in FOCUS) regarding her abilities to problem solve by instilling a belief that she is sufficiently skilled to solve the problem and instilling a sense of motivation. The training stresses the importance of realistic expectations about the time and effort that will be necessary to identify and use chosen strategies. The third component engages the client's “creativity” (“C” in FOCUS) by helping her brainstorm multiple solutions to the identified problem. Caregivers are instructed to think of as many solutions to the problem as possible and to write each solution down on a worksheet. The fourth component involves “understanding” (“U” in FOCUS) the patient's preference in considering which solutions to implement. Before deciding on a solution, the caregiver is encouraged to consider the potential outcomes of the chosen solutions and weigh the cost and benefits of each. The caregiver rates the likelihood that she will implement each solution as 0 (not at all likely), 1 (somewhat likely), or 3 (very likely). The fifth component is the implementation of the “solution” (“S” in FOCUS) and evaluation of how effective the solution was in solving the problem. This self-monitoring component is important to promote understanding about what made the chosen solution effective or ineffective and how to implement similar or alternative solutions in future problem situations.

The intervention was delivered by a registered nurse, whose own training was based on a training manual. The nurse was trained by the principal investigator using a one-on-one review of the intervention and was required to demonstrate the ability to deliver the intervention in a group of three caregivers who were not included in the study. The nurse and the principal investigator met monthly to discuss the intervention process and activities, in order to prevent intervention drift. Each intervention session was tape-recorded to ensure delivery of the intervention. The nurse also documented the amount of time spent weekly in delivering the intervention. Overall, the nurse completed the modules in 48 weeks (range, 130 to 150 minutes per module). Although the intervention module was intended to last for 105 minutes per module, the average amount of time spent to deliver the intervention was 138 minutes (S.D. = 11.36) per module.

Treatment fidelity related to receipt of the training intervention was determined by comparing baseline scores on a measure of resourcefulness, the SCS, with scores obtained immediately after intervention and 12 weeks after the intervention in family caregivers who participated in the intervention (*n* = 46). Among those who received training, mean scores on the SCS showed an increase in resourcefulness. Paired* t*-tests showed significant improvements in resourcefulness from baseline to 6 weeks (*t*(1, 49) = −3.62, *P* < 0.001), although no significant increase was found from baseline at 12 weeks ((*t*(1, 46) = −1.17, *P* = 0.25). Similar improvements were not seen in the caregivers who received the standard care at 6 weeks *t*(1, 44) = 1.50, *P* = 0.14) or at 12 weeks (*t*(1, 36) = 1.023, *P* = 0.31). Thus, the fidelity of the resourcefulness training intervention was demonstrated immediately after the training, although the improvement in resourcefulness was not sustained at 12 weeks after the intervention.

### 2.5. Comparison Group

A comparison group of family caregivers was assigned to standard care. After they completed the baseline data collection, these caregivers received a binder that included information on community resources, handouts about AD, and new scientific findings. Data were collected 6 weeks after baseline and 12 weeks after the second data collection period. With these families, there was no discussion on health-related matters or coping strategies before, during, or after the data collection.

### 2.6. Data Analysis

Descriptive statistics, including means and standard deviations, were examined for resourcefulness as well as for outcome variables of interest to determine the shape of distribution at baseline and after intervention. Correlations among the study variables were examined to determine relationships among these variables. Between group differences for primary and secondary outcome measures were analyzed. The baseline value corresponding to each response variable was used as a covariate in univariate analyses. The adjusted means were used for calculation of effect sizes [38]. Effect sizes were calculated to assess the magnitude of the effects of the intervention on the dependent variables. Effect size was calculated by subtracting the mean of the control group from the mean of the intervention group and then dividing by the pooled standard deviation [39]. Values of 0.2, 0.5, and 0.8 were defined as small, medium, and large effects, respectively [39]. A meaningful positive effect size was defined as one that was both greater than 0.20 and in favor of the intervention.

## 3. Results

Of the 138 family caregivers screened, 19 (14%) were ineligible and 14 (10%) did not want to participate. Of the remaining 105 who underwent baseline assessment, three changed their minds and decided not to participate and 102 (97%) were randomly assigned to the intervention group (50) or to the control group (52). Of the 50 family caregivers assigned to the intervention group, 49 (98%) completed the resourcefulness training and the second data collection interview; 46 (92%) completed the third interview. One caregiver died, one experienced declines in health, and two institutionalized the person with dementia. In the control group, only 44 (84%) of 52 family caregivers completed the second data collection interview; 36 (69%) of family caregivers completed the third interview. Five caregivers lost interest in the study, four institutionalized the person with dementia, three relocated out of state, and four did not continue caregiving.

The attrition rate was significantly higher in the control group than in the intervention group. There were no differences between those who stayed in the study and those who did not with regard to caregiver demographic characteristics such as race, age, and perceived health. However, there were differences in caregivers' years of education and relationship to the person with dementia. Caregivers with less than high school education were more likely to drop out of the study than were caregivers with high school or higher education. Additionally, nonspouse caregivers had a higher dropout rate than spouse caregivers. Family caregivers in the resourcefulness training and the usual-care group did not differ from each other at baseline in race (*χ* ^2^ = 1.18, *P* = 0.31), age (*t*(1,100) = −1.44, *P* = 0.15), household income (*χ* ^2^ = 1.33, *P* = 0.57), number of hours per week spent in caregiving (*t*(1, 100) = 1.69, *P* = 0.09), length of time in caregiving (*t*(1, 100) = 0.136, *P* = 0.89), relationship to the care recipient (*χ* ^2^ = 3.65, *P* = 0.09), or number of chronic conditions reported (*t* = −51, *P* = 0.61).

Table 1 presents the demographic and clinical baseline variables by group. The sample was composed of 102 female caregivers (97%) including 58 African Americans (57%) and 44 whites (43%). Their ages ranged from 35 to 85 years, with a mean age of 60 years. Persons with dementia ranged in age from 54 to 95 years, with a mean age of 80 years (standard deviation = 9.02 years).

On average, family caregivers reported having two chronic conditions. The most frequently reported conditions were high blood pressure (*N* = 41; 40%), arthritis (*N* = 26; 25%), diabetes (*N* = 16; 16%), emotional problems (*N* = 16; 16%), asthma (*N* = 11; 11%), circulatory conditions (*N* = 8; 8%), and cancer (*N* = 6; 6%). Despite their health problems, the majority of family caregivers (*N* = 79; 77%) perceived their health to be good or excellent. Anxiety scores (mean = 36 S.D. = 11.8) and depression scores (mean = 13.7 S.D. = 9.9) indicated that caregivers were experiencing low levels of anxiety and depression. Caregiver preparedness scores (mean = 2.4 S.D. = 0.80) indicated that they were fairly well prepared to care for the person with dementia.

Table 2 presents correlations on resourcefulness and outcome measures: depression, anxiety, preparedness, strain, mutuality, reward, and frequency of behavior problems. Resourcefulness was inversely related to anxiety and depression (*P* < 0.00), positively related to role reward (*P* < 0.00) and preparedness (*P* < 0.002).

Table 3 presents family caregivers' baseline data on primary and secondary outcomes. The groups did not differ in their baseline measures on primary outcomes of caregivers' learned resourcefulness (*t* = −1.01, *P* = 0.37) and depression (*t* = 1.61, *P* = 0.07). However, there was a significant difference in anxiety (*t* = 2.56, *P* < 0.03) between the groups. Specifically, at baseline, family caregivers in the control group reported higher anxiety symptoms compared with caregivers in the intervention group. The groups did not differ in their baseline measures on secondary outcomes of preparedness (*t* = 1.36, *P* = 0.19), mutuality (*t* = 0.33, *P* = 0.74), role reward (*t* = 0.078, *P* = 0.93), or role strain (*t* = −0.066, *P* = 0.94) or in the frequency of behavior problems in the person with dementia (*t* = 0.10, *P* = 0.92).

Table 4 presents adjusted means, standard deviations, and effect sizes for primary and secondary outcomes at weeks 6 and 12.

At week 6 (immediately after intervention), family caregivers in the intervention group reported significantly more resourcefulness skills (*F*(1, 91) = 13.54, *P* < 0.001) with a medium effect (0.54) compared with family caregivers in the control group. Family caregivers in the intervention group showed significantly higher anxiety symptoms at 6 weeks (immediately after intervention) compared with caregivers in the control group (*F*(1,91) = 4.90, *P* < 0.042), with an effect size of 0.44. Family caregivers in the intervention group did not differ significantly on caregiving preparedness from the control group (*F*(1,91) = 0.817, *P* = 0.14), with an effect size of 0.26. There were no immediate effects on mutuality, reward, strain, or frequency in occurrence of behavior problems.

At 12 weeks after intervention, there was a significant difference between groups on resourcefulness (*F*(1, 80) = 3.58, *P* = 0.048) although the effect size was small (0.36). Anxiety decreased in family caregivers in the intervention group (mean = 36.9) whereas it increased in the control group (mean = 39.7), although the difference was not significant ((*F* = 1.713, 80, *P* = 0.194) and the effect size was small (0.29). There was no treatment effect and effect size was less than 0.2 for depression. Caregivers in the intervention group showed higher scores in preparedness compared with the control group (*F*(1, 80) = 3.37, *P* = 0.041), with an effect size of 0.41. There were no treatment effects and effect sizes were less than 0.2 for secondary outcomes on mutuality, reward, role strain, and frequency in occurrence of behavior problems.

All family caregivers in the intervention program reported that the intervention was helpful for them and for the person with dementia. As one caregiver reported, “It helped me to understand how Alzheimer's disease affects my husband's behavior. I used to take everything he said personally.” Another caregiver said, “You would all be so proud of me, I called the Alzheimer's Association and talked to the staff to ask about resources in my community. I would never have done this if not for this program because I did not know a thing about Alzheimer disease.”

Many participants in the control group reported that they benefited “some” from participating in the study. This finding may indicate that even the task of responding to questionnaires during the data collection at baseline, 6 weeks, and 12 weeks had positive effects on caregivers, perhaps because they felt they were being helpful by participating in research.

## 4. Discussion

This study demonstrated that positive effects on role outcome (preparedness) and anxiety can be achieved in family caregivers of persons with dementia through use of the interventions described here. The group intervention was successful in increasing resourcefulness in caregivers immediately at 6 weeks and in decreasing anxiety in caregivers and increasing caregiver preparedness at 12 weeks after the intervention. The findings also showed that for resourcefulness to be sustained, a booster dose in training may be needed.

This study represents the first attempt to examine the immediate and extended (over 12 weeks) effects of teaching resourcefulness on caregiver role outcomes (preparedness and mutuality), emotional outcomes (anxiety and depression), and patient outcome (frequency of behavior problems). Although baseline data revealed that greater resourcefulness was significantly associated with lower anxiety and depression, the intervention was found to have no effect in reducing depression. This lack of effect may be due to the caregivers' relatively low scores on depression upon study enrollment. Because they were not depressed initially, there was little room for improvement. In contrast, the intervention appeared to make a difference in caregivers' anxiety. Caregivers in the intervention group reported increased anxiety symptoms compared with control-group caregivers at 6 weeks. This outcome is surprising because we expected that anxiety would decrease as resourcefulness was enhanced. The increase in caregivers' anxiety in the intervention group may have resulted from their newly acquired knowledge about the disease and the uncertainty of the condition. This new knowledge may heighten the caregiver's anticipation and feelings of loss as they understand more about the disease and the course of decline that it will follow. This result is consistent with the findings of Graham et al. [40] and Proctor et al. [41], indicating that knowledge about AD increases anxiety. Another possible reason for the increase in anxiety in caregivers in the intervention group at 6 weeks may be the timing of data collection, which coincided with the completion of the intervention program. Family caregivers who participated in the intervention group developed a sense of belonging and support. The termination of the program may have triggered anxiety and a sense that they now had to move forward by themselves. Although a session was devoted to stress management, relaxation exercises need to be reinforced in each session. However, at 12 weeks after intervention, family caregivers' anxiety decreased, while it increased in the control group.

The findings also showed a significant difference in preparedness between the groups at 3 months after the intervention. The delayed effects on caregiver preparedness at 12 weeks after the intervention suggest that preparedness skills may take time to be fully integrated into a caregiver's repertoire. As a consequence, when caregiver preparedness increased 12 weeks after the intervention, their feelings of anxiety decreased. The findings support the self-regulation theory that providing educational information about AD to family caregivers facilitated the formation of a cognitive schema that strengthened caregivers' understanding about disease-associated changes, how to interpret these changes, and how to adapt to them. Additionally, the findings partially support Rosenbaum's conceptual model of learned resourcefulness [42], suggesting that the skills constituting resourcefulness can be taught in interactions with others. In addition, the positive effects of learned resourcefulness training found in this study are consistent with Rosenbaum's view of the beneficial effects of learned resourcefulness on the regulation of emotions. The findings also support the need to continue to assess family caregiver preparedness.

## 5. Implications

There are two important implications from this study. First, study results demonstrate that family caregivers can be taught resourcefulness successfully in a small group in the community. Caregivers in the training group demonstrated increased resourceful skills. The study suggests that “knowing more” about AD may lead to anxiety. Providing educational program about Alzheimer's disease must be coupled with support and skill-building training. Resourcefulness as a training program was delivered in a group form that provided support to family caregivers and involves problem solving, cognitive reframing, and learning self-regulation of emotional response (depression and anxiety). This training will benefit family caregivers who are anxious, depressed, less prepared, or less resourceful. Second, there is a need to assess family caregiver's preparedness when they assume the role of caregivers for person with dementia. The delayed effects on caregiver preparedness at 12 weeks after the intervention suggest that caregiver preparedness may take time to be fully integrated into a caregiver's repertoire. Family caregivers' who are prepared are less anxious.

Several limitations of this pilot study include the small sample size and the exclusion of male caregivers. The small sample size limited the power to detect significant differences between the study groups on some of the patient and caregiver outcomes.

Attrition is a potential problem in studies in which outcome measures are obtained over time. We addressed this challenge by recruiting more participants than needed for the desired statistical analysis. In addition, we may have reduced the attrition rate by calling caregivers on the phone as a reminder for the upcoming intervention session and conducting home visits rather than mailing questionnaires in the follow-up phase. In this study, we used ano-treatment condition as a control, which included provision of standard materials because that is the only “intervention” that the vast majority of caregivers in the population receive. This lack of provision of treatment may account for more attrition in this group. In future larger studies, it will be appropriate to include a credible attention-placebo control.

Despite these limitations, the results of this study provide support for the effectiveness of the intervention program and its theoretical framework. Findings from this study hold the promise that persons with dementia and their family caregivers can benefit from a theoretically driven, reproducible intervention aimed at improving the caregivers' resourcefulness.

## Figures and Tables

**Table 1:** Demographic and clinical baseline variables by group.

Variables	Intervention group (*n* = 50)	Usual-care group (*n* = 52)	*P* value
Family caregivers			
Mean age (SD)	61.91 (11.91)	58.46 (11.99)	.15
Race			
White	24 (48%)	20 (38%)	.31
African American	26 (52%)	32 (62%)	
Household income (yearly)			
<$14,000	5 (13%)	5 (10%)	.39
$15,000–24,999	1 (3%)	11 (24%)	
$25,000–44,999	16 (34%)	14 (29%)	
≥$50,000	16 (34%)	19 (37%)	
Education∗			
Below high school	1 (2%)	4 (8%)	.37
High school	8 (12%)	17 (34%)	
Some college	16 (37%)	13 (26%)	
Completed college	9 (21%)	10 (20%)	
Completed graduate education	7 (16%)	4 (8%)	
Marital status∗			
Married	29 (63%)	25 (48%)	.11
Widowed	2 (4%)	11 (21%)	
Divorced	5 (11%)	8 (15%)	
Separated	1 (2%)	1 (2%)	

Relationship to care recipient			
Spouse	16 (31%)	8 (15%)	.09
Nonspouse	34 (69%)	44 (85%)	

Care recipients			
Mean age	78.64 (9.37)	81 (8.64)	.18
Sex			
Male	22 (44%)	21 (40%)	.85
Female	28 (56%)	31 (60%)	
Race			
White	24 (48%)	21 (40%)	.42
African American	26 (52%)	31 (60%)	.62
Number of care recipient illnesses (mean)	2.8 (1.39)	2.93 (1.01)	.61
Number of caregiver illnesses (mean)	1.4 (1.4)	1.3 (1.2)	.11
Total hours/week on caregiving	17.31 (14.72)	26.15 (12.5)	.09

*Missing data.

**Table 2:** Correlations of resourcefulness on outcome measures.

Outcome variables	*r*	*P* value
State anxiety	−.37	.000
Depressive symptoms	−.33	.001
Preparedness	.32	.002
Mutuality	.16	.61
Role reward	.39	.001
Role strain	.05	.62
Frequency of behavior problems	−.07	.50

**Table 3:** Family caregivers' baseline data for dependent variables.

Variables	Intervention group (*n* = 50)	Usual-care group (*n* = 52)	*P* value
Resourcefulness∗	41.13 (23.0)	36.49 (27.72)	.37
State anxiety∗	33.83 (11.30)	38.91 (11.82)	.03
Depressive symptoms∗	11.85 (7.25)	15.41 (11.69)	.07
Preparedness^+^	2.29 (.77)	2.51 (.89)	.19
Mutuality^+^	2.45 (.89)	2.51 (.81)	.74
Role reward^+^	2.76 (1.01)	2.78 (.92)	.93
Role strain^+^	1.99 (.71)	1.97 (.95)	.94
Frequency of behavior problems^+^	1.38 (.68)	1.39 (.69)	.92

*Primary outcome.

^+^Secondary outcome.

**Table 4:** Adjusted means, standard deviations, and effect sizes for study variables over time.

Variables^a^	Resourcefulness training group (*n* = 50), mean (SD)	Usual-care group (*n* = 52), mean (SD)	Effect size	*P* value
Resourcefulness∗				
T2	46.47 (27.37)	31.80 (27.37)	.54	<.001
T3	43.32 (28.83)	33.04 (28.83)	.36	.048
Depression∗				
T2	14.49 (8.89)	14.07 (8.89)	.05	.77
T3	13.23 (8.90)	14.41 (8.90)	.13	.13
Anxiety∗				
T2	40.72 (12.21)	35.33 (12.21)	.44	.042
T3	36.99 (11.97)	39.70 (11.97)	.23	.29
Preparedness∗				
T2	2.58 (.74)	2.39 (.74)	.26	.14
T3	2.86 (.70)	2.57 (.70)	.41	.041
Role reward^+^				
T2	2.94 (.85)	2.89 (.85)	.07	.68
T3	3.00 (.90)	2.92 (.90)	.08	.61
Mutuality^+^				
T2	2.45 (.77)	2.52 (.77)	.09	.54
T3	2.76 (.79)	2.52 (.79)	.30	.10
Role strain^+^				
T2	1.94 (.83)	1.84 (.83)	.12	.43
T3	1.90 (.88)	1.85 (.88)	.06	.78
Behavior problems (frequency)^+^				
T2	1.31 (.64)	1.34 (.64)	.05	.83
T3	1.31 (.60)	1.62 (.60)	.11	.11

*Primary outcome.

^+^Secondary outcome

^a^Preintervention scores were used as covariates in the analysis.

T2 = 6 weeks (immediately after intervention); T3 = 12 weeks after intervention.

Values of .2, .5, and .8 were defined as small, medium, and large effect sizes, respectively.
